# Supplementary material for: Deep-sea-floor diversity in Asteroidea is shaped by competing processes across different latitudes and oceans
Source: Nat Ecol Evol. 2025 Aug 8;9(10):1910–23. doi: 10.1038/s41559-025-02808-2 (PMC12507690; doi:10.1038/s41559-025-02808-2)
Supplement: Supplementary file 1 — Supplementary Table 1 and Figs. 1–3. [file 41559_2025_2808_MOESM1_ESM.pdf]

nature ecology & evolution

Supplementary information https://doi.org/10.1038/s41559-025-02808-2

# Deep-sea-floor diversity in Asteroidea is shaped by competing processes across different latitudes and oceans

In the format provided by the authors and unedited

| N° | Realm | Marine Eco-province | Species | Genera | Families |
|---|---|---|---|---|---|
| 1 | Temperate Northern Pacific | Cold Temperate Northwest Pacific | 123 | 38 | 14 |
| 2 | Temperate Northern Pacific | Cold Temperate Northeast Pacific | 106 | 44 | 18 |
| 3 | Temperate Northern Pacific | Warm Temperate Southeast Pacific | 26 | 23 | 16 |
| 4 | Temperate Northern Pacific | Warm Temperate Northeast Pacific | 71 | 41 | 19 |
| 5 | Tropical Eastern Pacific | Tropical East Pacific | 59 | 34 | 18 |
| 6 | Tropical Eastern Pacific | Galapagos | 23 | 23 | 14 |
| 7 | Temperate Southern Africa | Benguela | 32 | 23 | 11 |
| 8 | Temperate Southern Africa | Agulhas | 60 | 38 | 14 |
| 9 | Tropical Atlantic | West African Transition | 47 | 26 | 11 |
| 10 | Tropical Atlantic | Gulf of Guinea | 38 | 21 | 9 |
| 11 | Temperate Northern Atlantic | Mediterranean | 31 | 18 | 10 |
| 12 | Temperate Northern Atlantic | Northern European Seas | 53 | 36 | 17 |
| 13 | Temperate Northern Atlantic | Lusitanian | 45 | 32 | 16 |
| 14 | Temperate South America | Warm Temperate Southwest Atlantic | 47 | 36 | 16 |
| 15 | Temperate South America | Magellanic | 62 | 42 | 17 |
| 16 | Tropical Atlantic | St Helena and Ascension Islands | 17 | 13 | 7 |
| 17 | Temperate Northern Atlantic | Cold Temperate Northwest Atlantic | 50 | 32 | 14 |
| 18 | Temperate Northern Atlantic | Warm Temperate Northwest Atlantic | 70 | 39 | 17 |
| 19 | Tropical Atlantic | Tropical Northwestern Atlantic | 86 | 47 | 20 |
| 20 | Tropical Atlantic | North Brazil Shelf | 42 | 24 | 14 |
| 21 | Tropical Atlantic | Tropical Southwestern Atlantic | 30 | 16 | 10 |
| 22 | Western Indo-Pacific | Somali/Arabian | 59 | 35 | 14 |
| 23 | Western Indo-Pacific | Red Sea and Gulf of Aden | 53 | 31 | 13 |
| 24 | Western Indo-Pacific | West Indian Ocean | 103 | 57 | 23 |
| 25 | Western Indo-Pacific | Central Indian Ocean Islands | 59 | 36 | 14 |
| 26 | Western Indo-Pacific | West and South Indian Shelf | 70 | 37 | 14 |
| 27 | Western Indo-Pacific | Bay of Bengal | 40 | 23 | 11 |
| 28 | Central Indo-Pacific | Java Transitional | 90 | 43 | 15 |
| 29 | Central Indo-Pacific | Sunda Shelf | 111 | 51 | 17 |
| 30 | Western Indo-Pacific | Andaman | 111 | 51 | 15 |
| 31 | Central Indo-Pacific | Western Coral Triangle | 187 | 84 | 26 |
| 32 | Central Indo-Pacific | Northeast Australian Shelf | 114 | 56 | 17 |
| 33 | Central Indo-Pacific | Sahul Shelf | 121 | 51 | 19 |
| 34 | Central Indo-Pacific | Northwest Australian Shelf | 113 | 47 | 16 |
| 35 | Central Indo-Pacific | South China Sea | 106 | 55 | 21 |
| 36 | Central Indo-Pacific | Tropical Southwestern Pacific | 90 | 57 | 17 |
| 37 | Central Indo-Pacific | Eastern Coral Triangle | 82 | 52 | 18 |
| 38 | Central Indo-Pacific | Tropical Northwest Pacific | 70 | 41 | 16 |
| 39 | Central Indo-Pacific | South Kuroshio | 77 | 49 | 16 |
| 40 | Eastern Indo-Pacific | Marquesas | 10 | 8 | 4 |
| 41 | Eastern Indo-Pacific | South East Polynesia | 18 | 15 | 8 |
| 42 | Eastern Indo-Pacific | Central Polynesia | 24 | 18 | 10 |
| 43 | Eastern Indo-Pacific | Marshall, Gilbert and Ellis Islands | 30 | 18 | 10 |
| 44 | Eastern Indo-Pacific | Hawaii | 38 | 25 | 10 |
| 45 | Temperate Northern Pacific | Warm Temperate Northwest Pacific | 92 | 54 | 17 |
| 46 | Temperate Australasia | Southern New Zealand | 61 | 41 | 16 |
| 47 | Temperate Australasia | Northern New Zealand | 67 | 46 | 21 |
| 48 | Central Indo-Pacific | Lord Howe and Norfolk Island | 59 | 39 | 17 |
| 49 | Temperate Australasia | Southwest Australian Shelf | 67 | 42 | 15 |
| 50 | Temperate Australasia | Southeast Australian Shelf | 86 | 50 | 18 |
| 51 | Temperate Australasia | East Central Australian Shelf | 93 | 58 | 18 |
| 52 | Temperate Australasia | West Central Australian Shelf | 79 | 41 | 15 |

**Supplementary Table 1:** Shallow water diversity by eco-province. Number of species, genera and families reported from shallow water (0-200 m) based on coordinate and literature records. Marine eco-province numbers align with those used in Fig. 3B.

Global Shallow

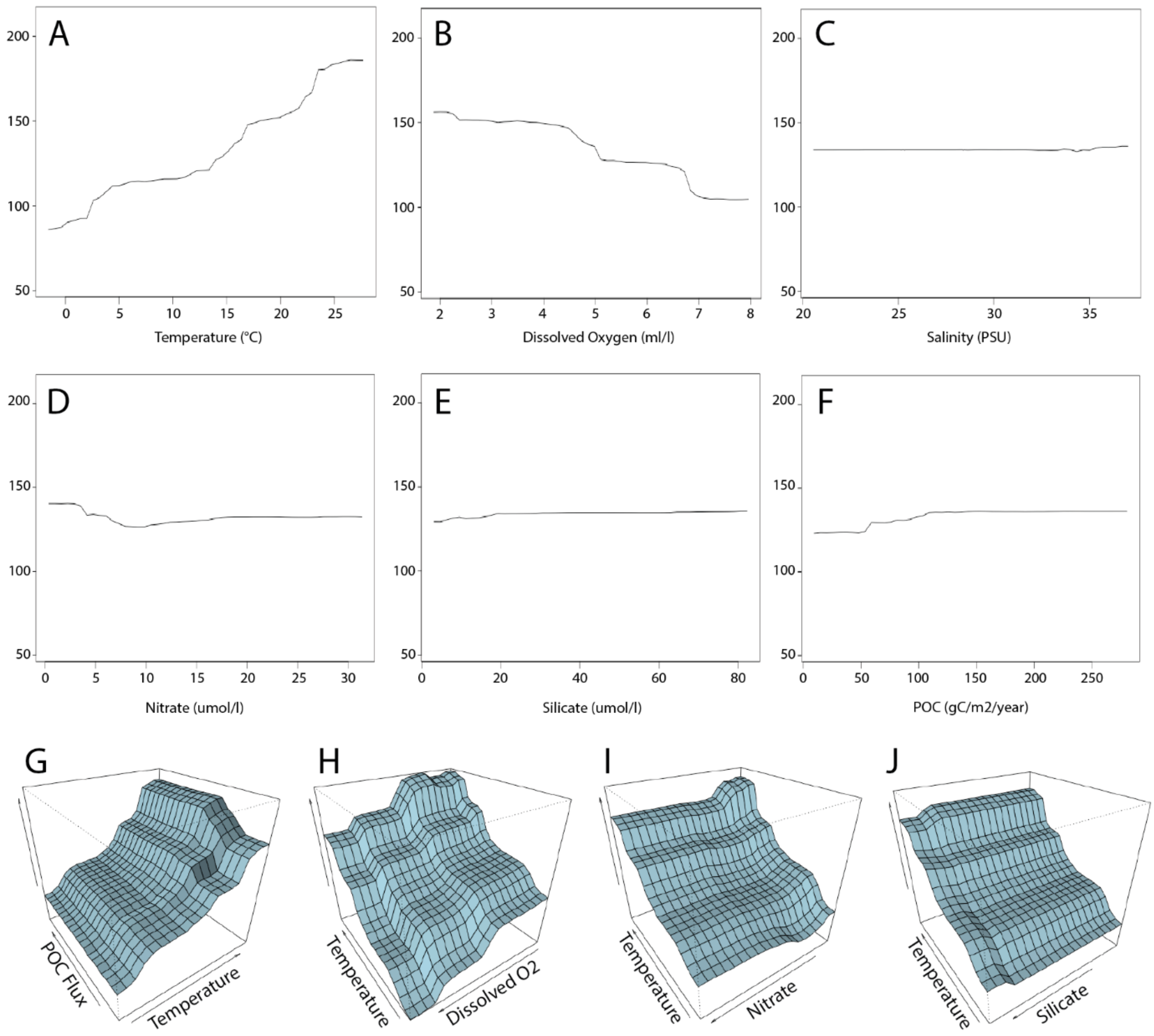

Global Bathyal

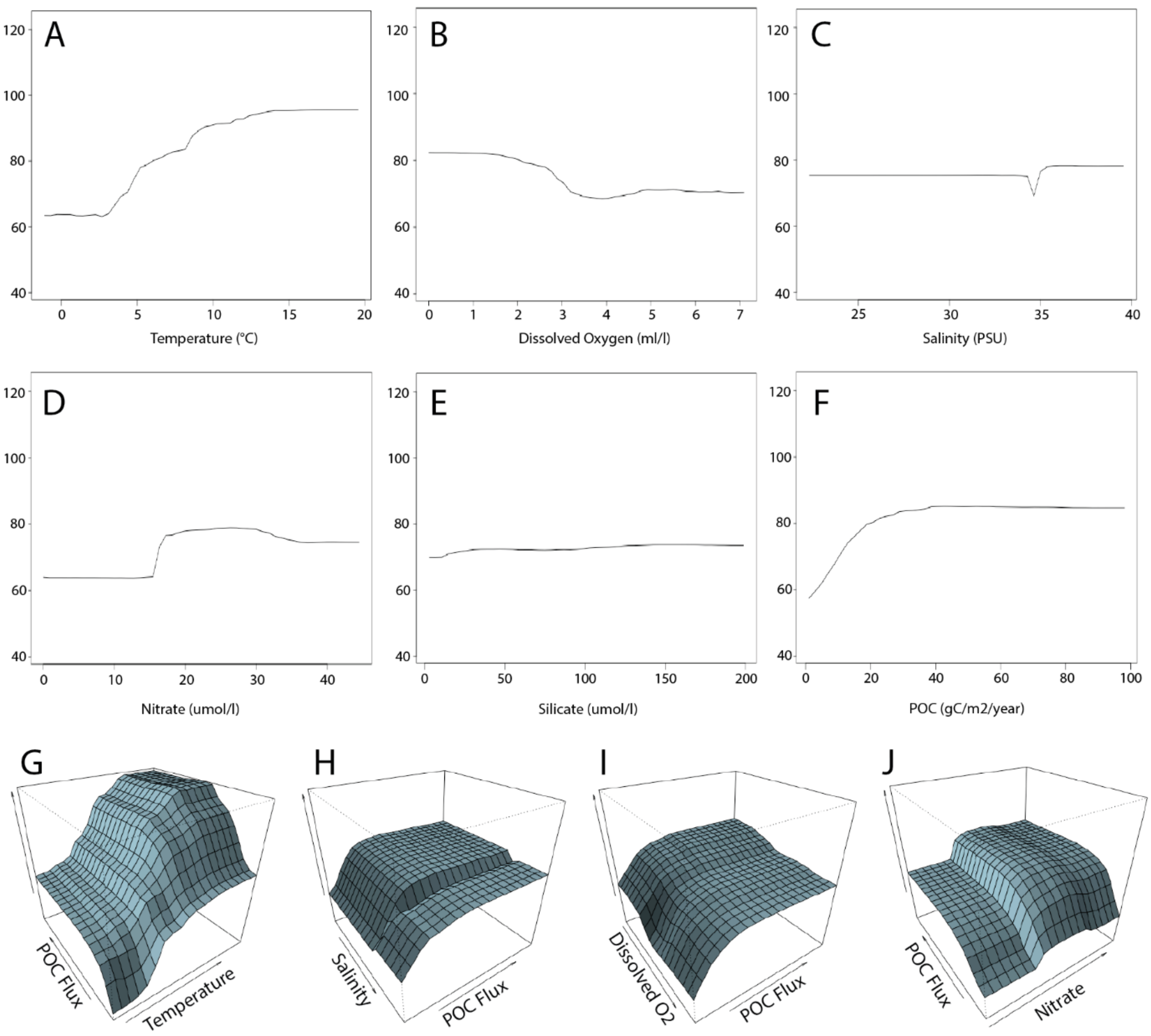

Global Lower Bathyal and Abyssal

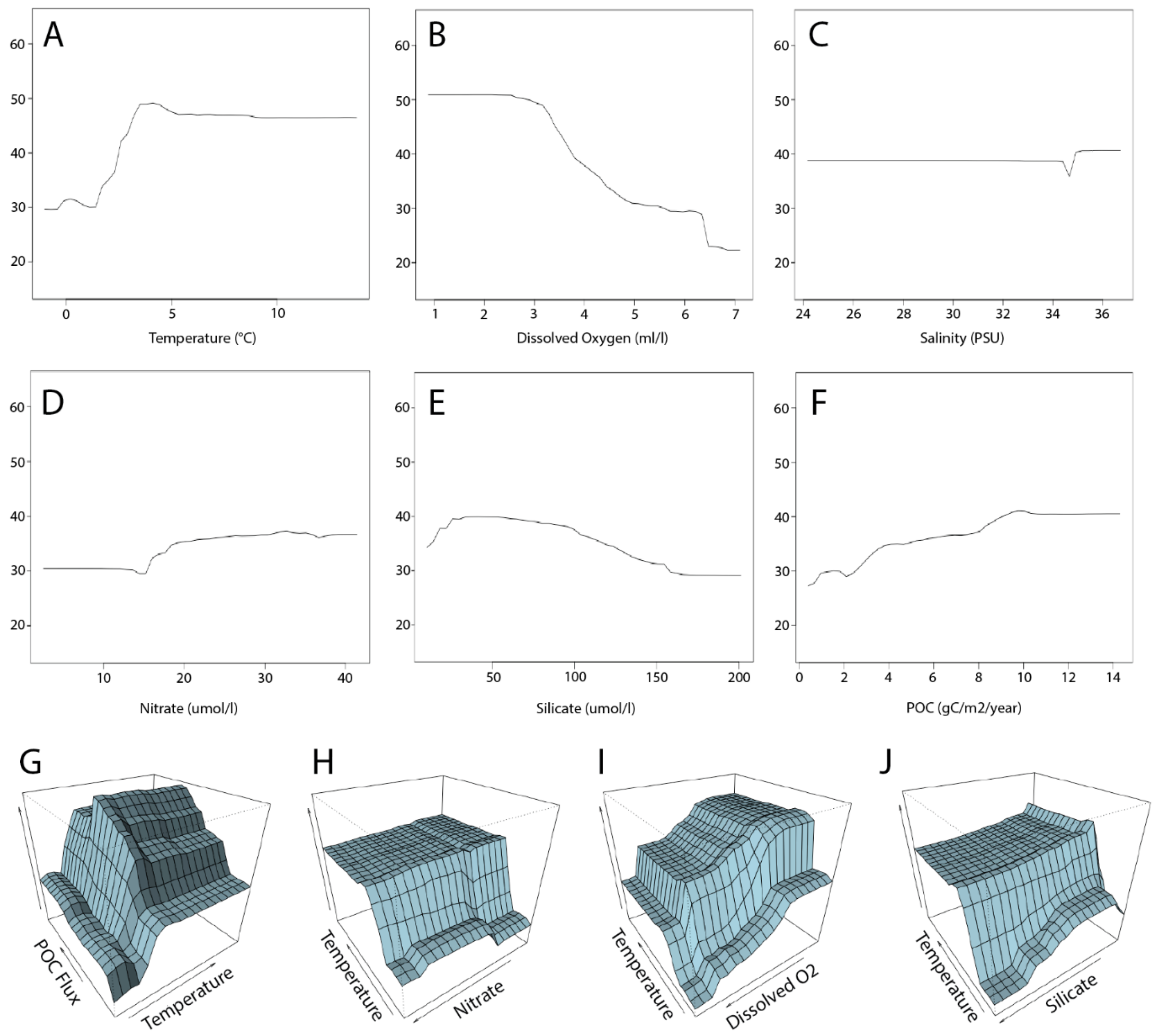

Northern Shallow

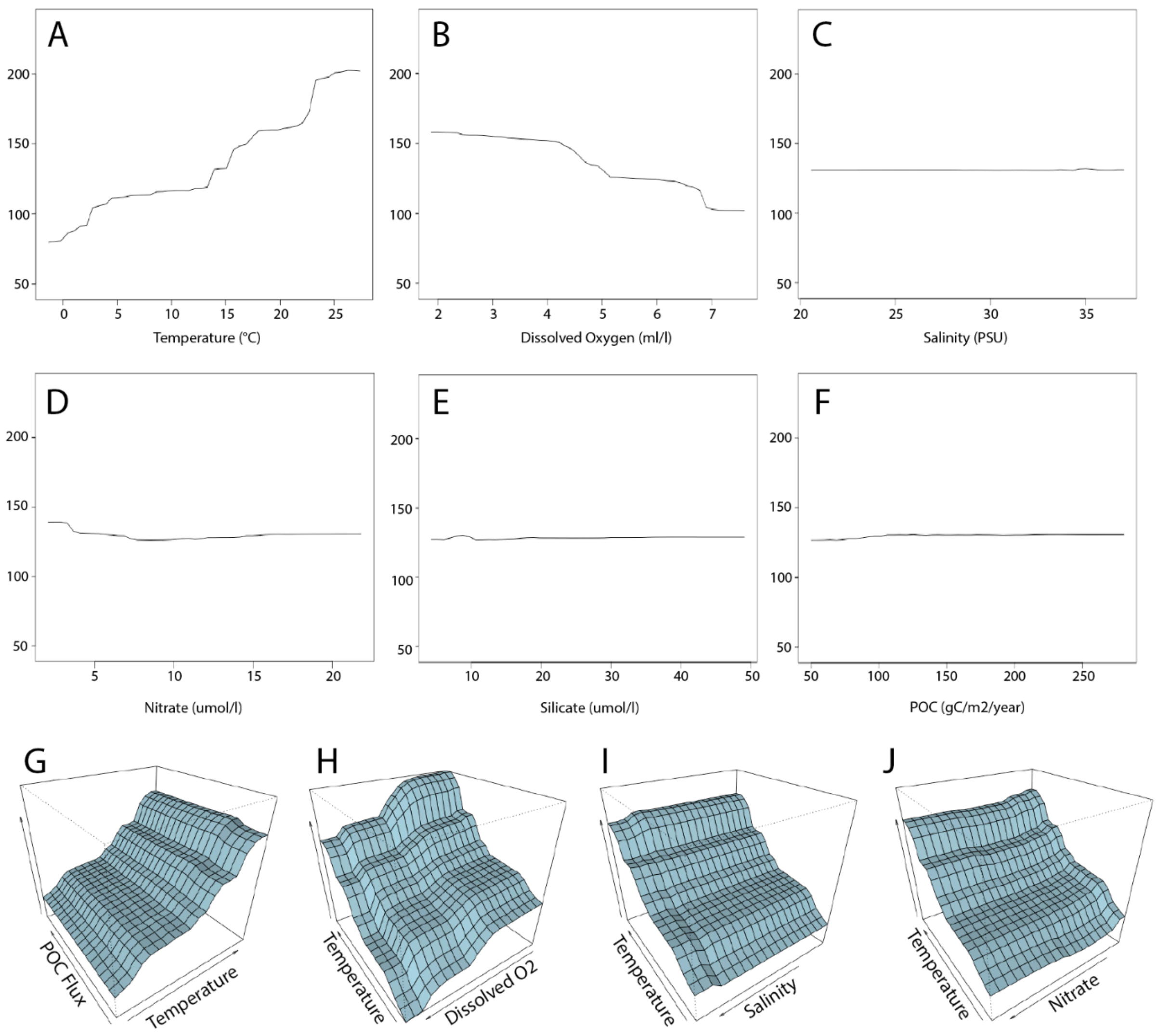

Northern Bathyal

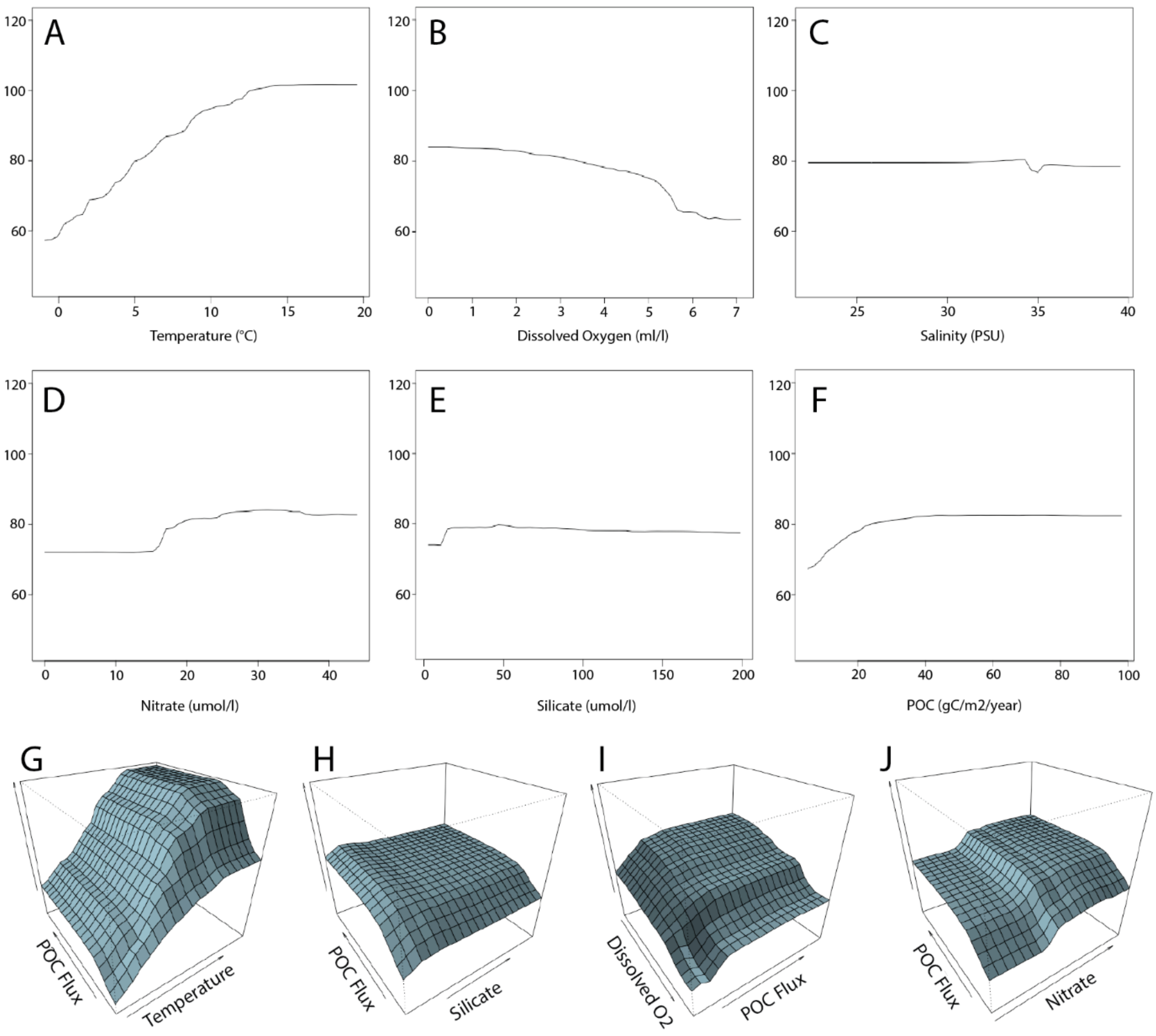

Northern Lower Bathyal and Abyssal

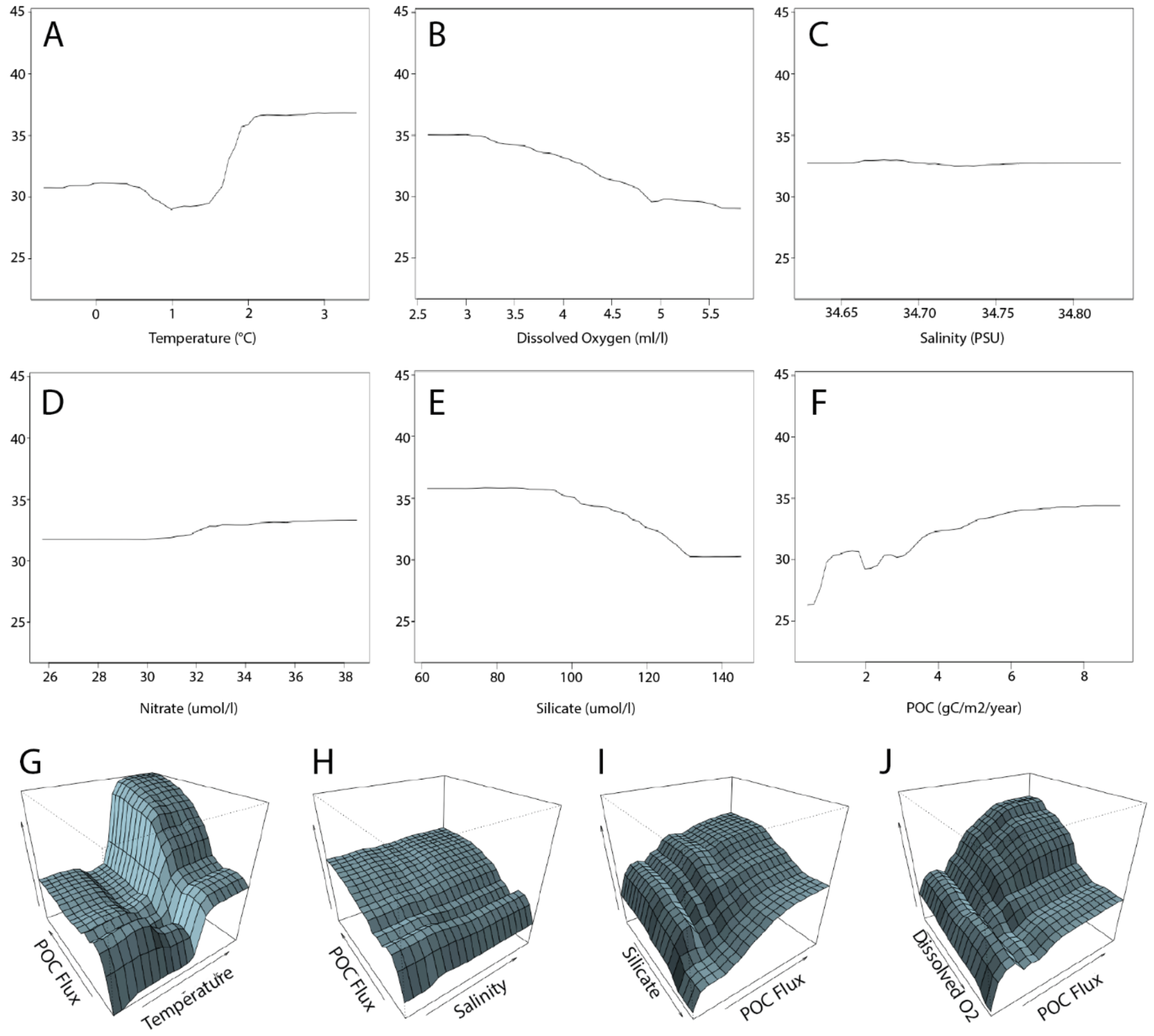

Southern Shallow

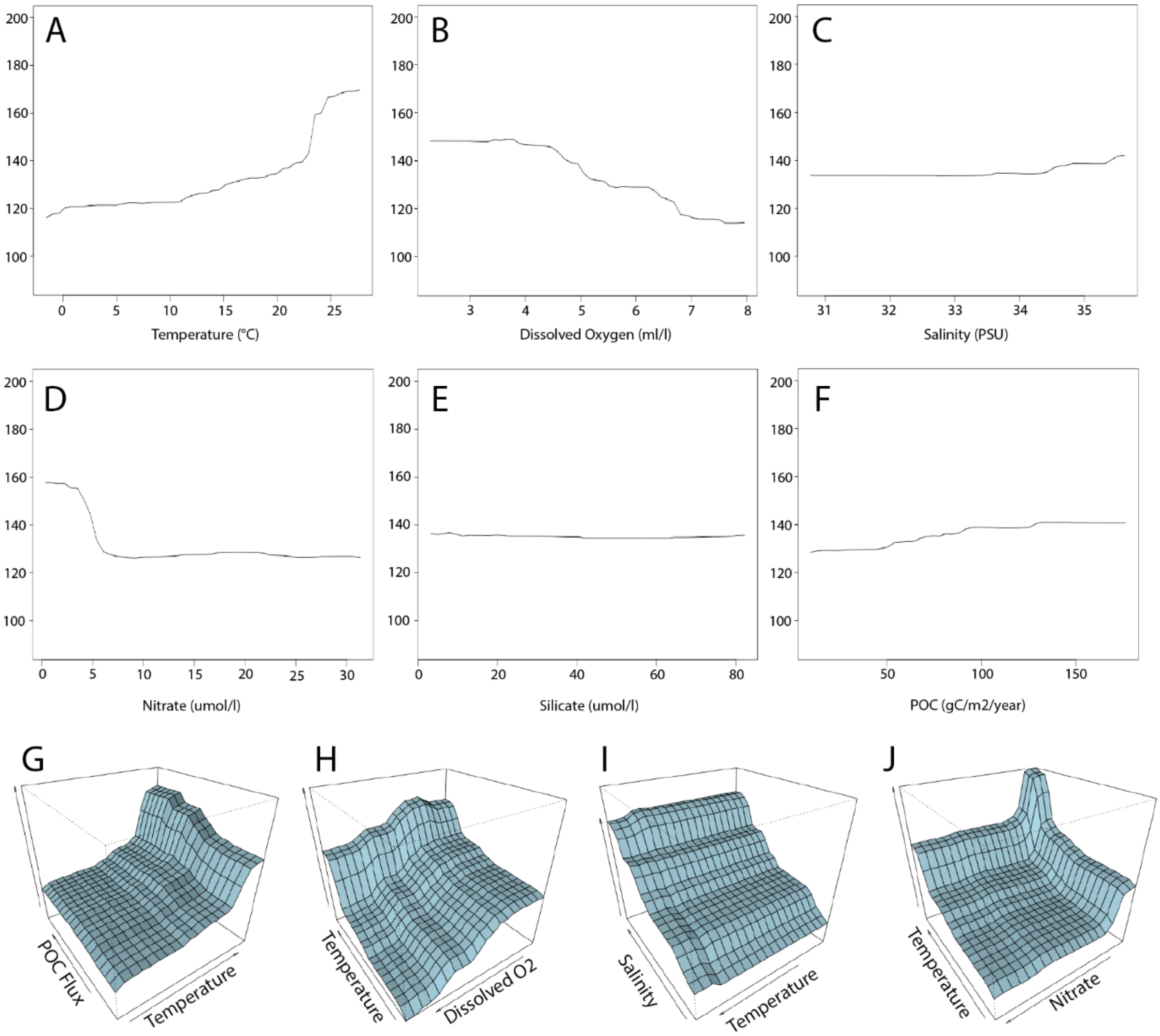

Southern Bathyal

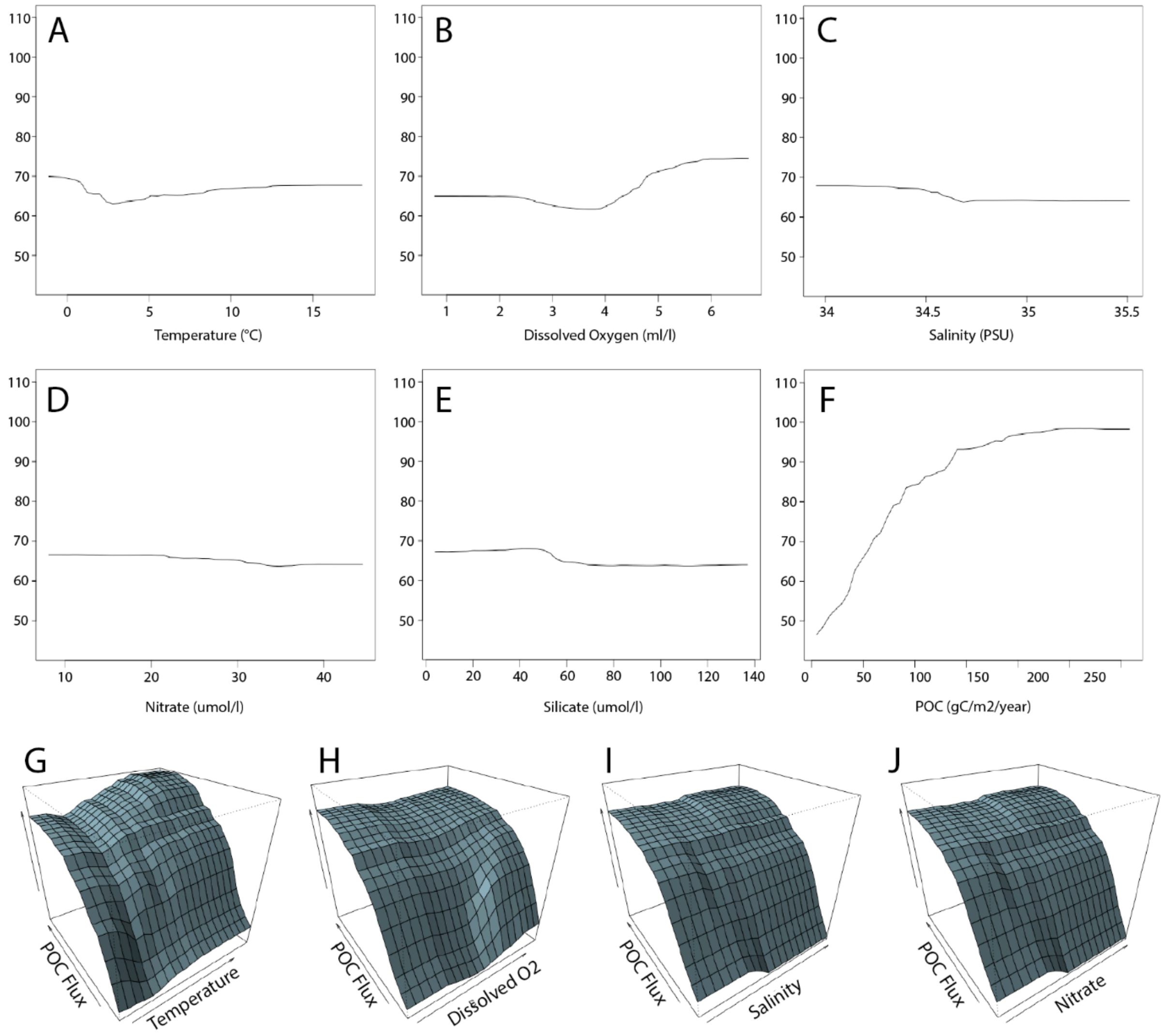

Southern Lower Bathyal and Abyssal

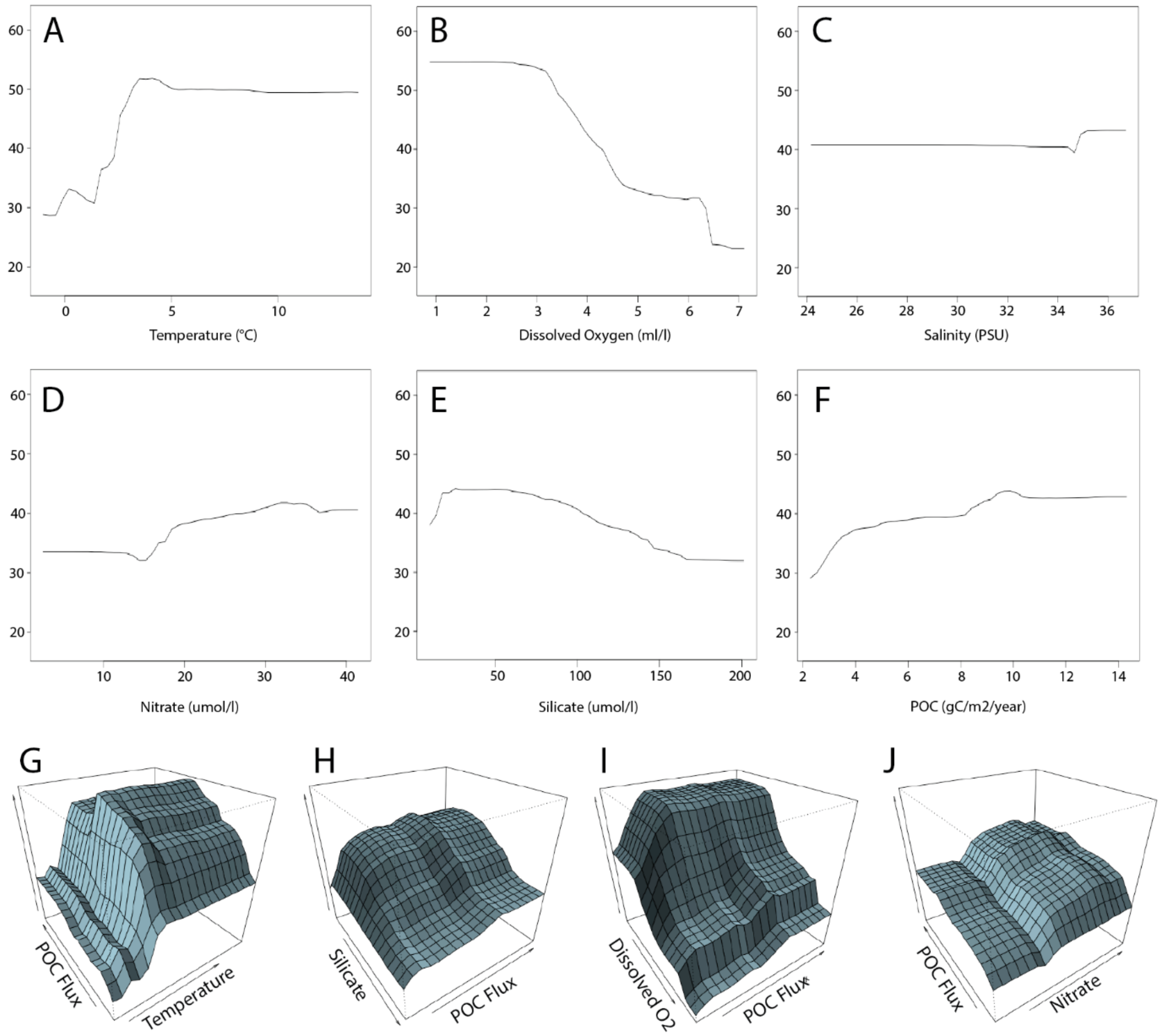

Atlantic Shallow

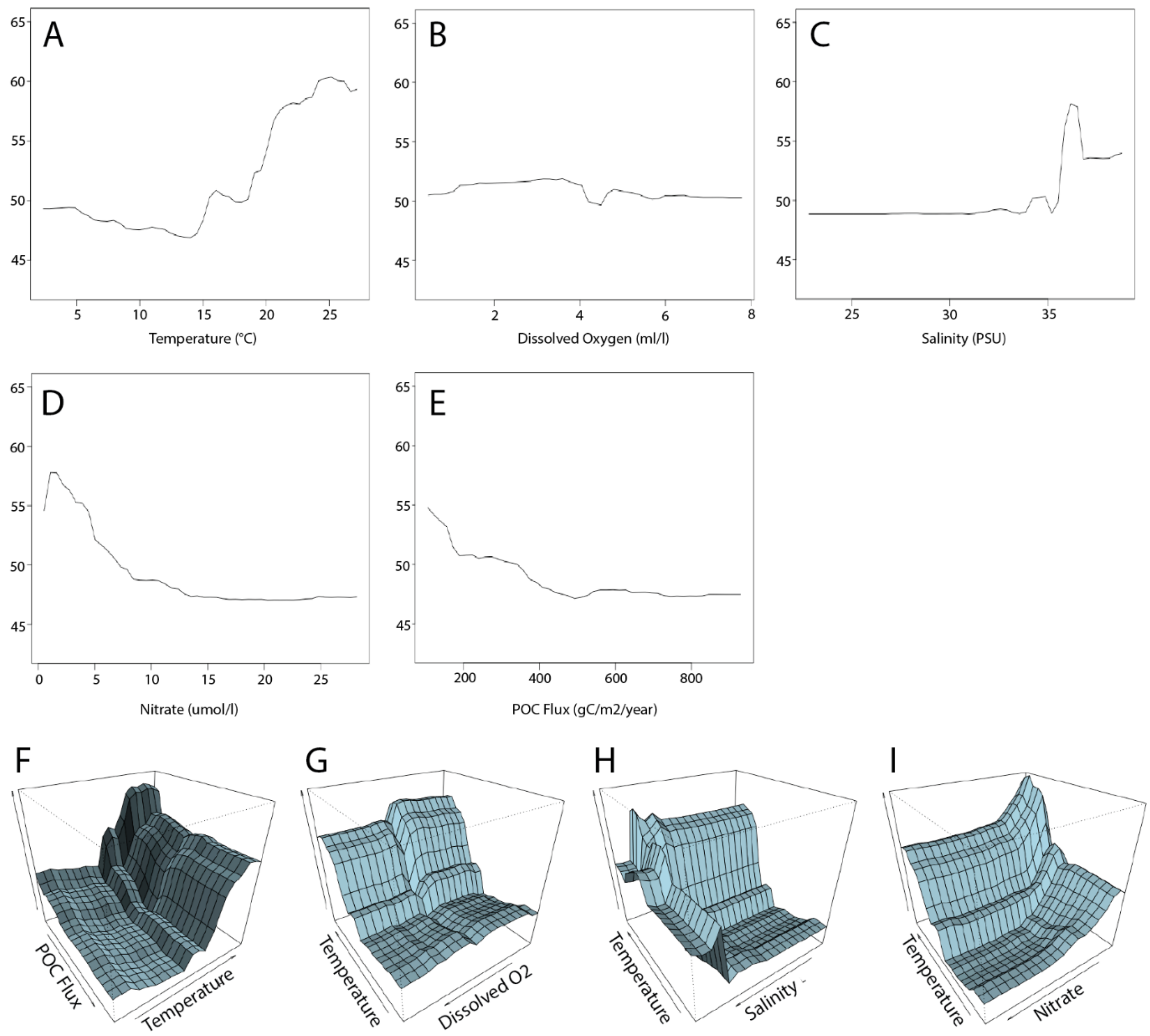

Atlantic Bathyal

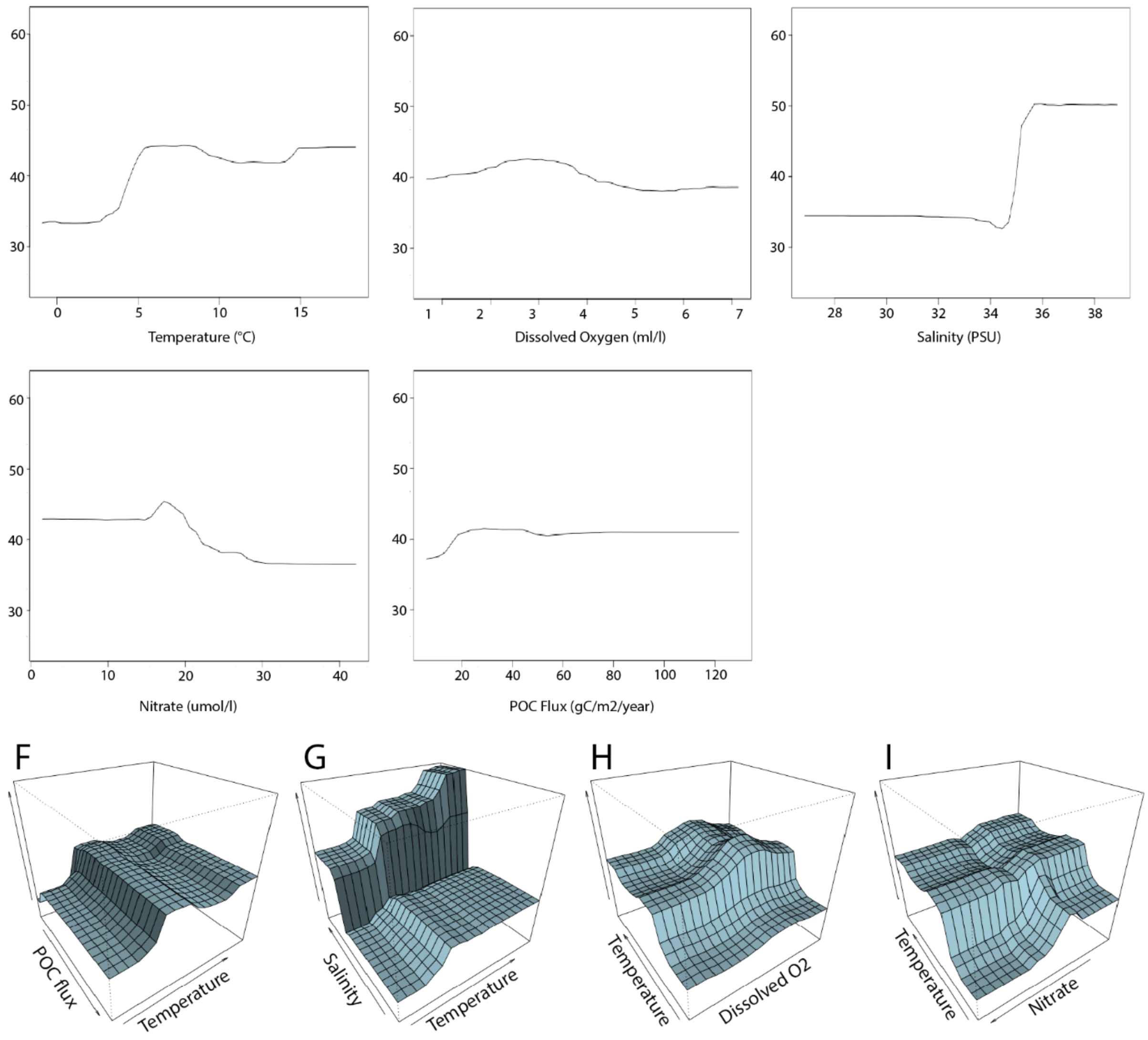

Atlantic Lower Bathyal and Abyssal

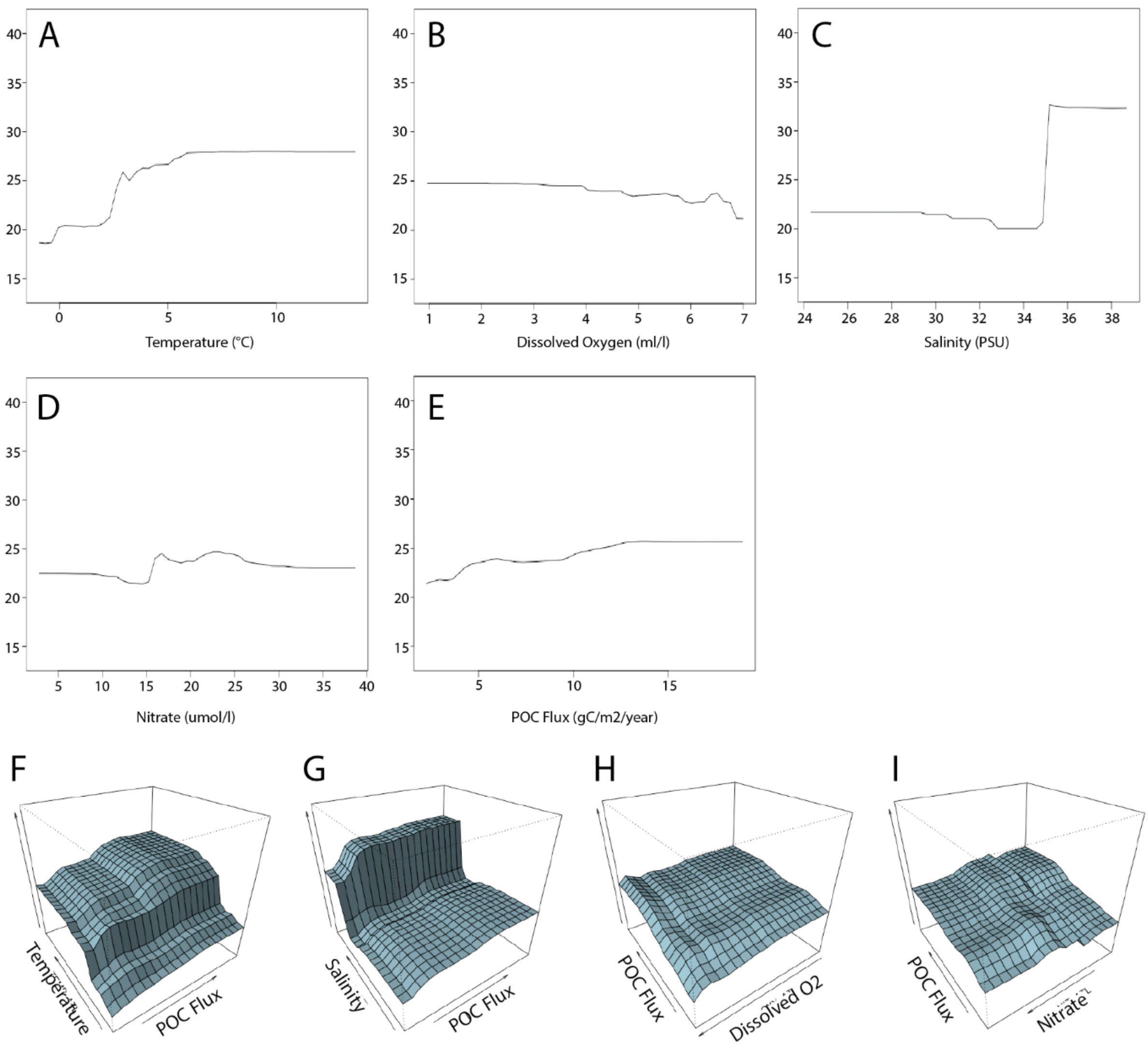

IWP Shallow

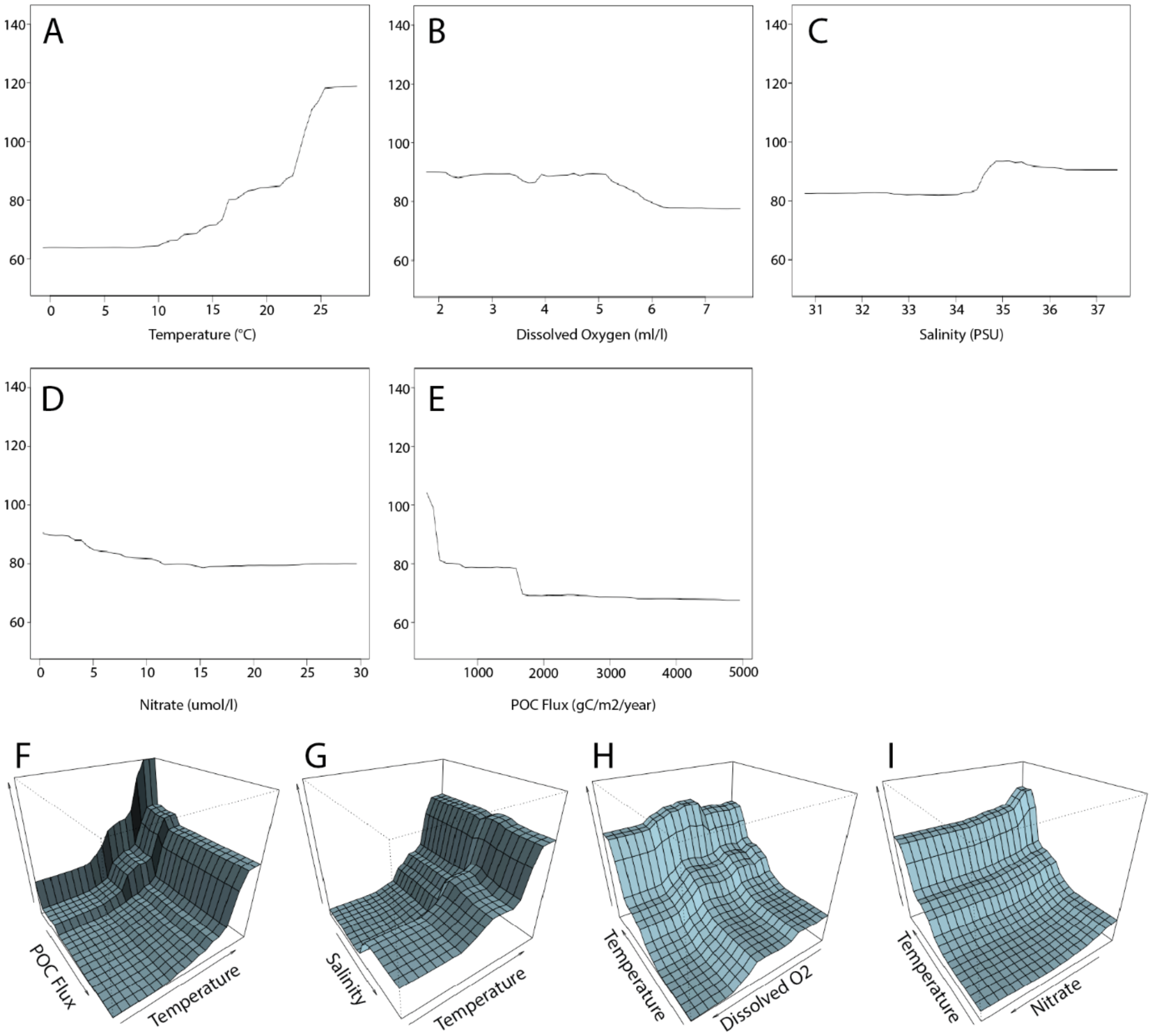

A
140
120
100
80
60
0
5
10
15
20
25
Temperature (°C)
B
2
3
4
5
6
7
Dissolved Oxygen (ml/l)
C
31
32
33
34
35
36
37
Salinity (PSU)
D
0
5
10
15
20
25
30
Nitrate (umol/l)
E
1000
2000
3000
4000
5000
POC Flux (gC/m2/year)
F
POC Flux
Temperature
G
Salinity
Temperature
H
Temperature
Dissolved O2
I
Temperature
Nitrate

IWP Bathyal

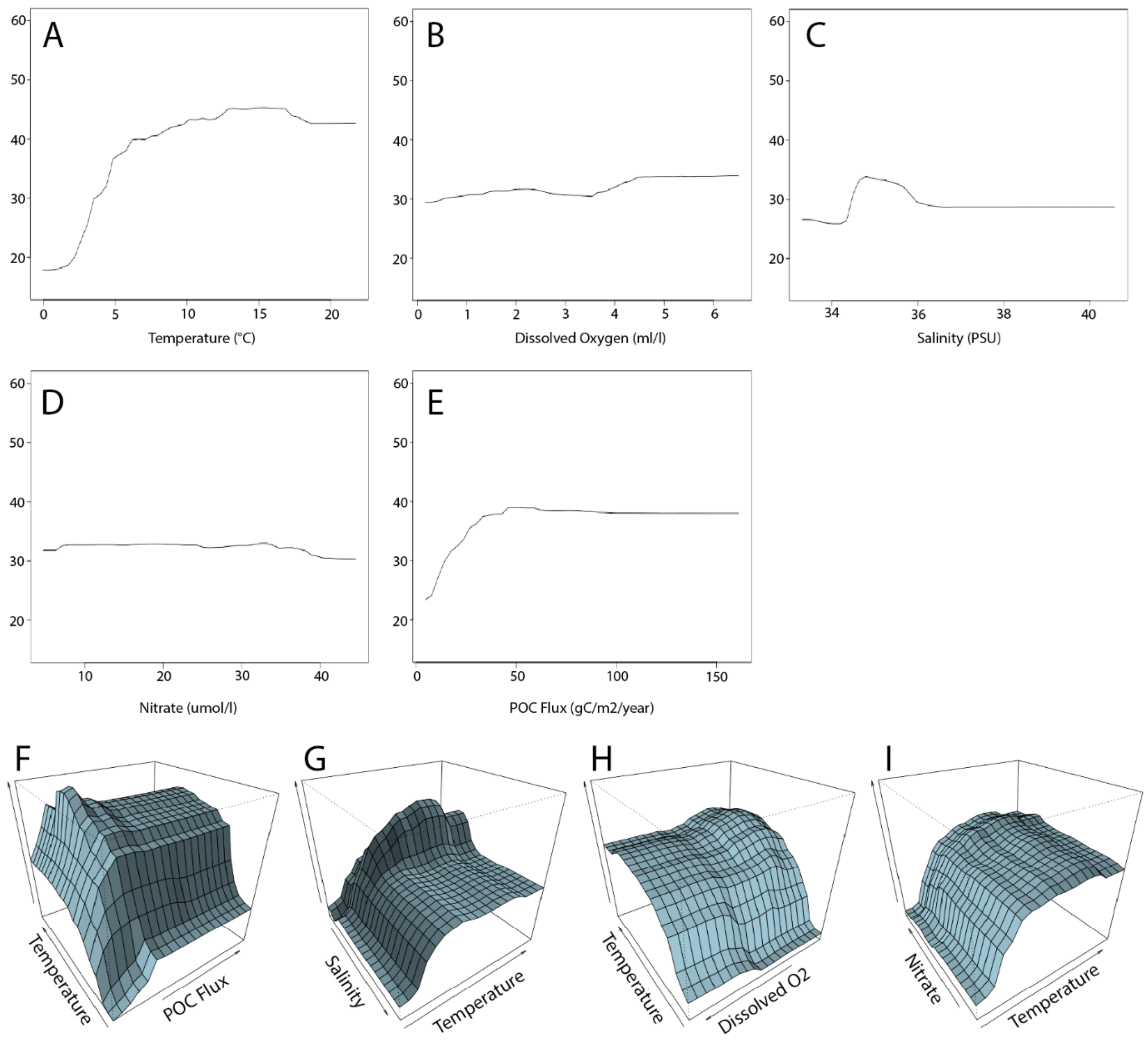
A
B
C
D
E
F
G
H
I
Temperature (°C)
Dissolved Oxygen (ml/l)
Salinity (PSU)
Nitrate (umol/l)
POC Flux (gC/m2/year)
Temperature
POC Flux
Salinity
Temperature
Temperature
Dissolved O2
Nitrate
Temperature

IWP Lower Bathyal and Abyssal

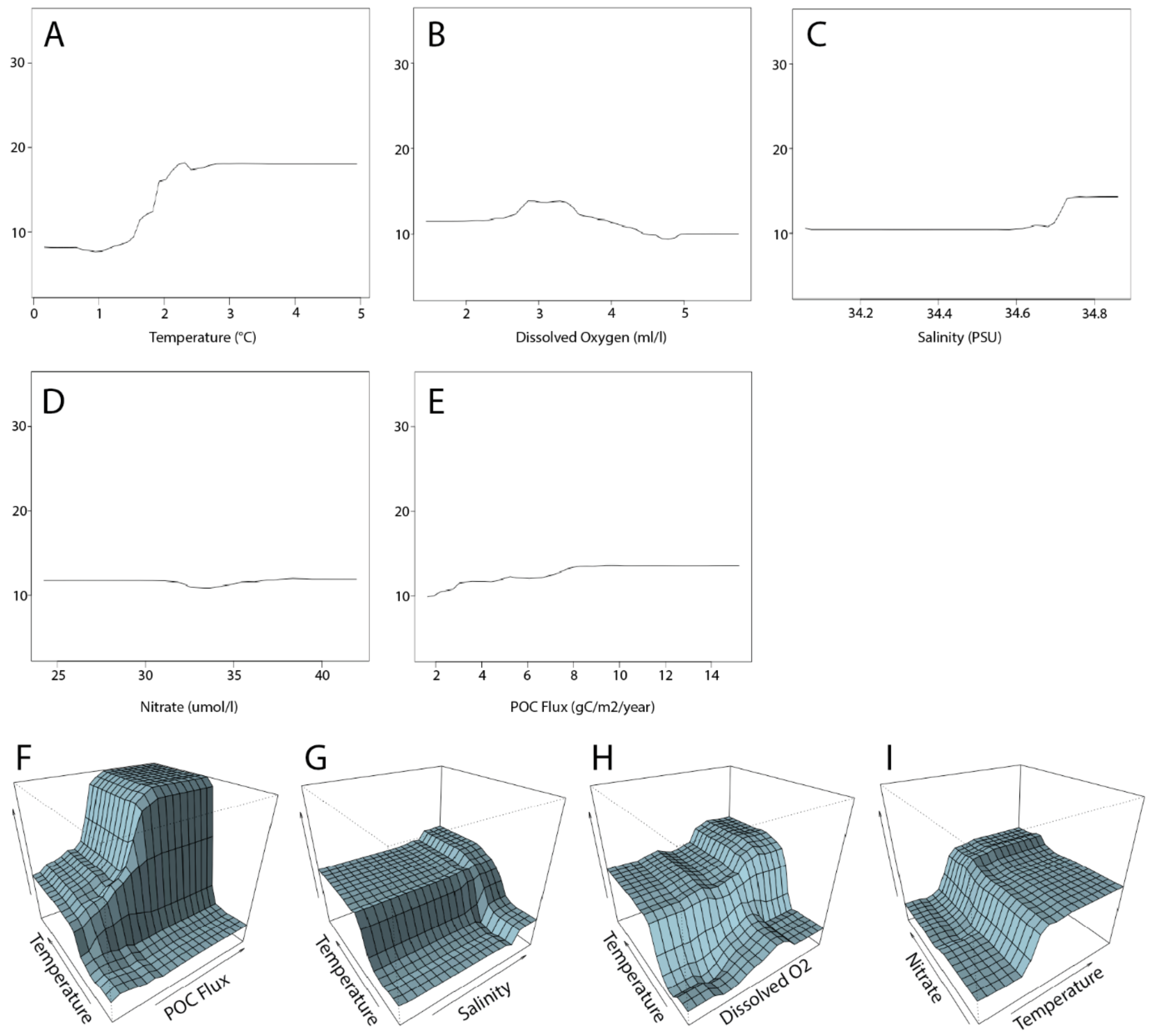


**Supplementary Figure 1:** Partial dependency plots for all Random Forest model outputs across fully interpolated data for the full global dataset and each of the hemispheric, basins and bathymetric zones (Shallow: 0-200 m; Upper Bathyal: 200 - 2,000 m; Lower Bathyal and Abyssal: 2,000 - 6,000 m) tested in this study. For the global and hemispheric plots (A-I), characters refer to the following: A) Partial dependency plots for Temperature; B) Partial dependency plots for Dissolved Oxygen; C) Partial dependency plots for Salinity; D) Partial dependency plots for Nitrates; E) Partial dependency plots for Silicates; F) Partial dependency plots for Particulate Organic Carbon (POC) flux; G-J) Pairwise interactions between the four most significant variable combinations across all model outputs.Arrows along axes display increasing levels of each variable. The vertical extent of each plot represents maximum species richness explained by the combined variables, with the upper limit defined as the total species richness estimated for each bathome. For the ocean basin plots (J-O), characters refer to the following: A) Partial dependency plots for Temperature; B) Partial dependency plots for Dissolved Oxygen; C) Partial dependency plots for Salinity; D) Partial dependency plots for Nitrates; E) Partial dependency plots for POC flux; F-I) Pairwise interactions between the four most significant variable combinations across all model outputs. Axes indicate increasing values of each variable as for plots A-I).

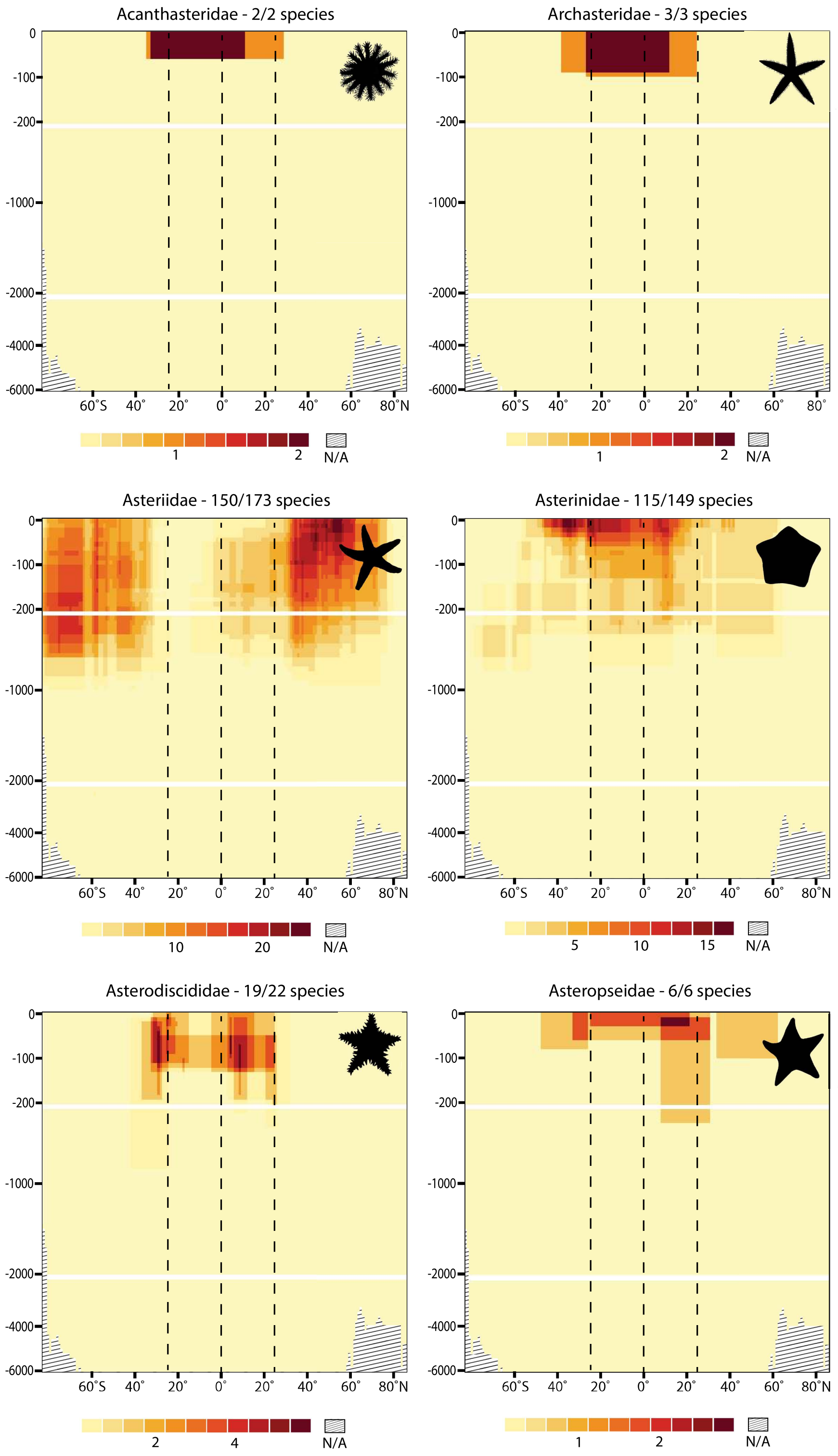
Acanthasteridae - 2/2 species
Archasteridae - 3/3 species
Asteriidae - 150/173 species
Asterinidae - 115/149 species
Asterodiscididae - 19/22 species
Asteropseidae - 6/6 species
0
-100
-200
-1000
-2000
-4000
-6000
60˚S
40˚
20˚
0˚
20˚
40˚
60˚
80˚N
N/A

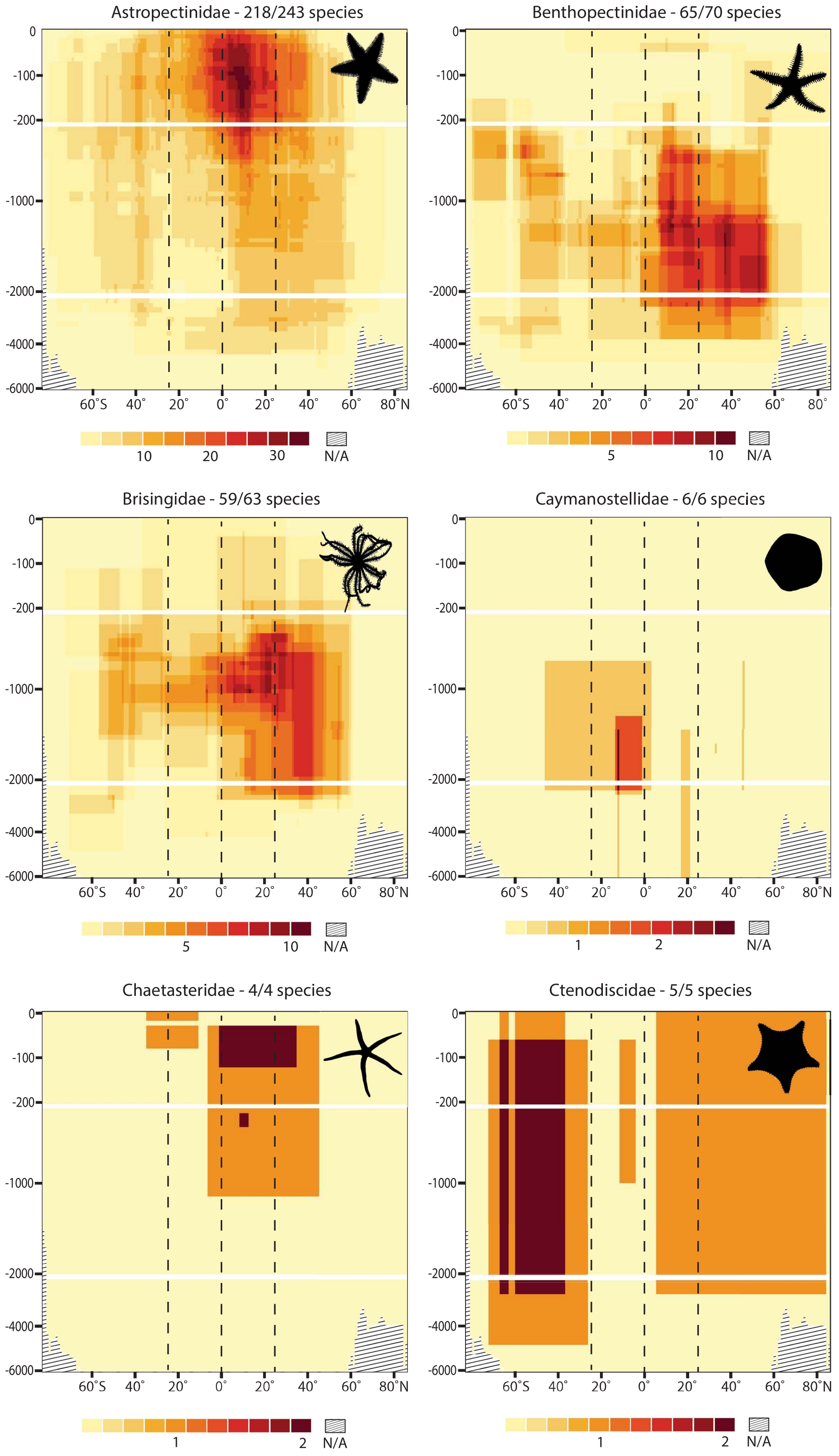

Astropectinidae - 218/243 species
0
-100
-200
-1000
-2000
-4000
-6000
60˚S
40˚
20˚
0˚
20˚
40˚
60˚
80˚N
10
20
30
N/A
Benthopectinidae - 65/70 species
5
10
N/A
Brisingidae - 59/63 species
5
10
N/A
Caymanostellidae - 6/6 species
1
2
N/A
Chaetasteridae - 4/4 species
1
2
N/A
Ctenodiscidae - 5/5 species
1
2
N/A

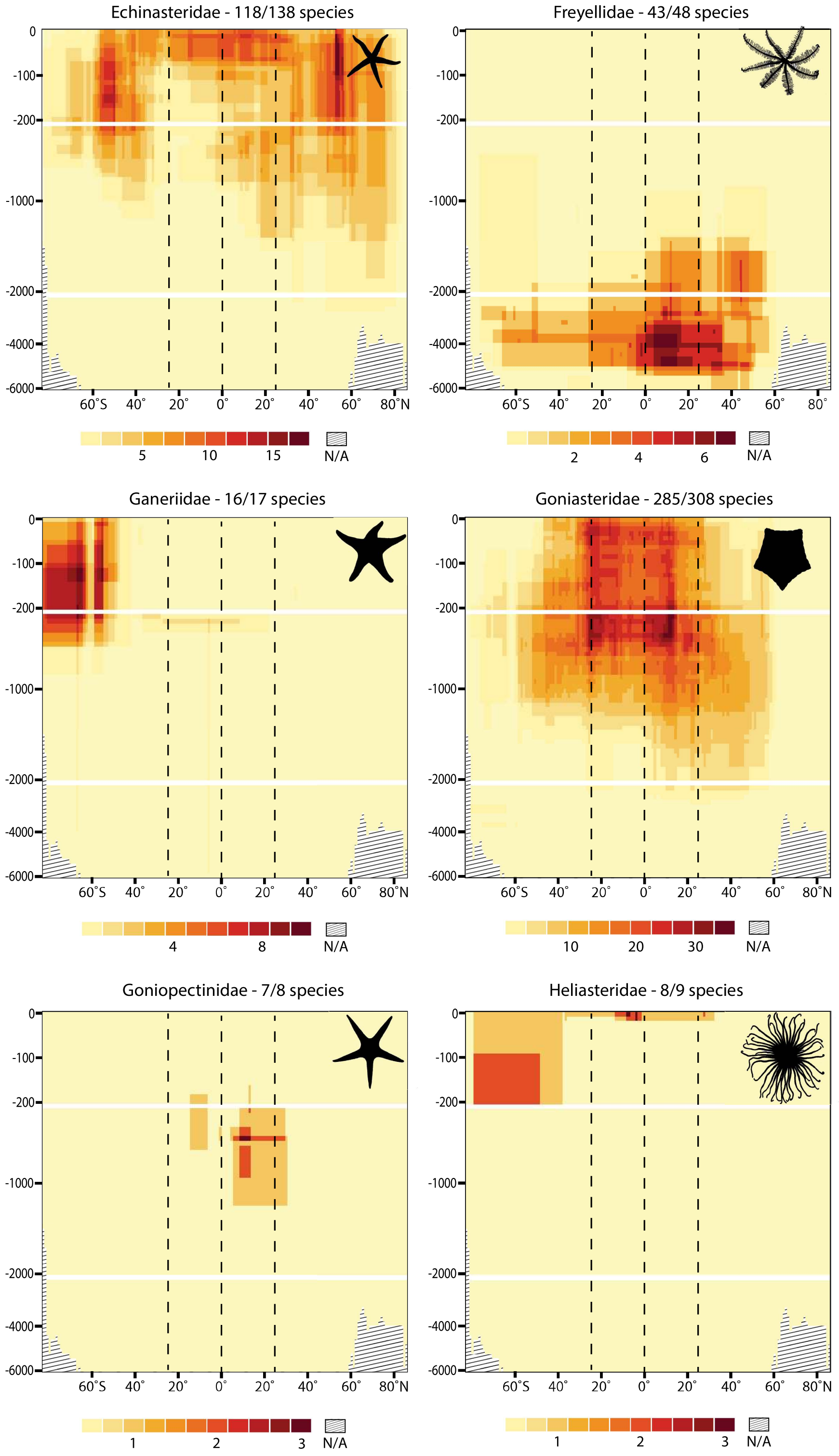

Echinasteridae - 118/138 species
Freyellidae - 43/48 species
Ganeriidae - 16/17 species
Goniasteridae - 285/308 species
Goniopectinidae - 7/8 species
Heliasteridae - 8/9 species
0
-100
-200
-1000
-2000
-4000
-6000
60°S
40°
20°
0°
20°
40°
60°
80°N
80°
5
10
15
2
4
6
8
20
30
1
3
N/A

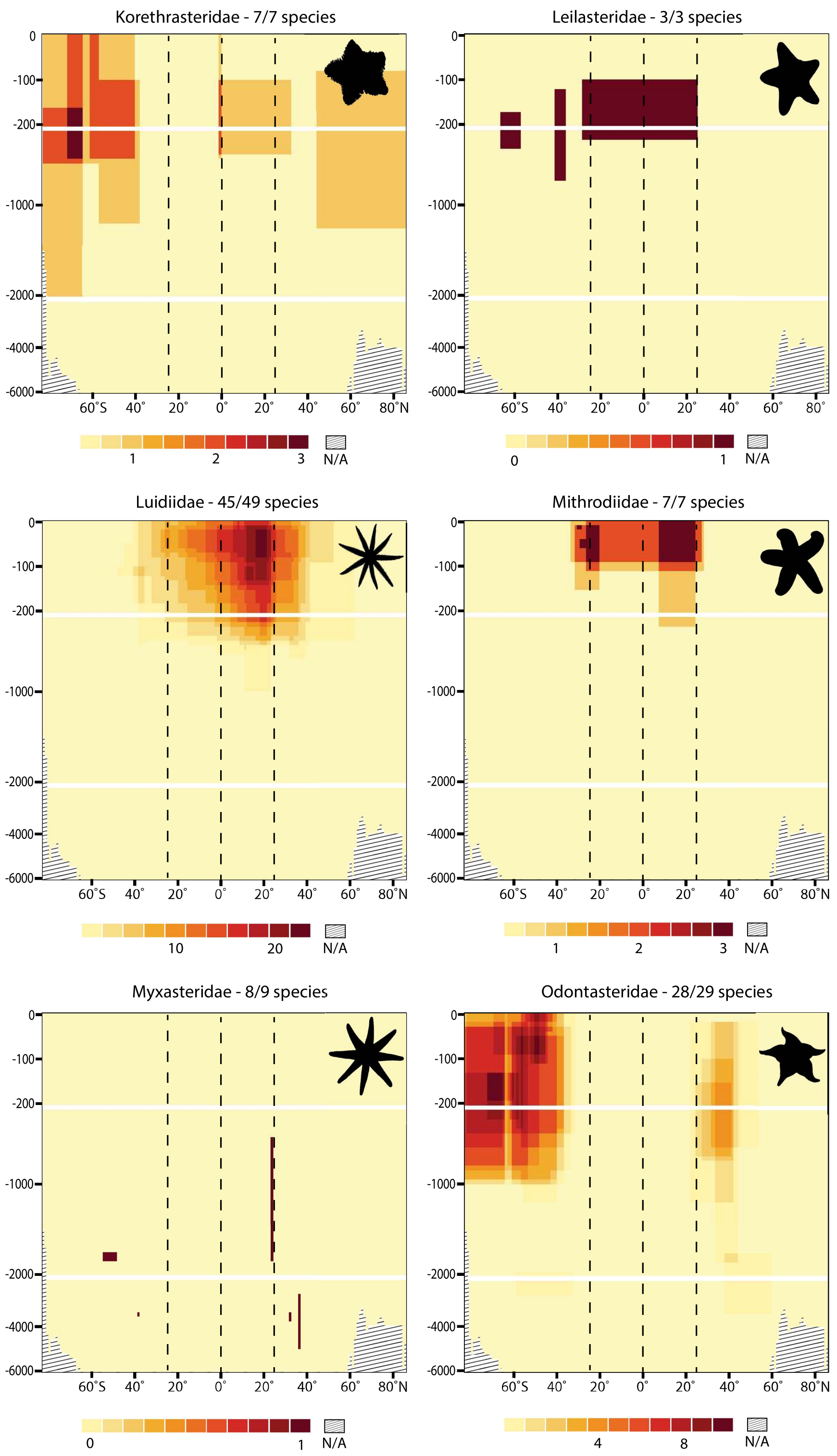

Korethrasteridae - 7/7 species
Leilasteridae - 3/3 species
Luidiidae - 45/49 species
Mithrodiidae - 7/7 species
Myxasteridae - 8/9 species
Odontasteridae - 28/29 species
0
-100
-200
-1000
-2000
-4000
-6000
60˚S
40˚
20˚
0˚
20˚
40˚
60˚
80˚N
80˚
1
2
3
0
10
20
4
8
N/A

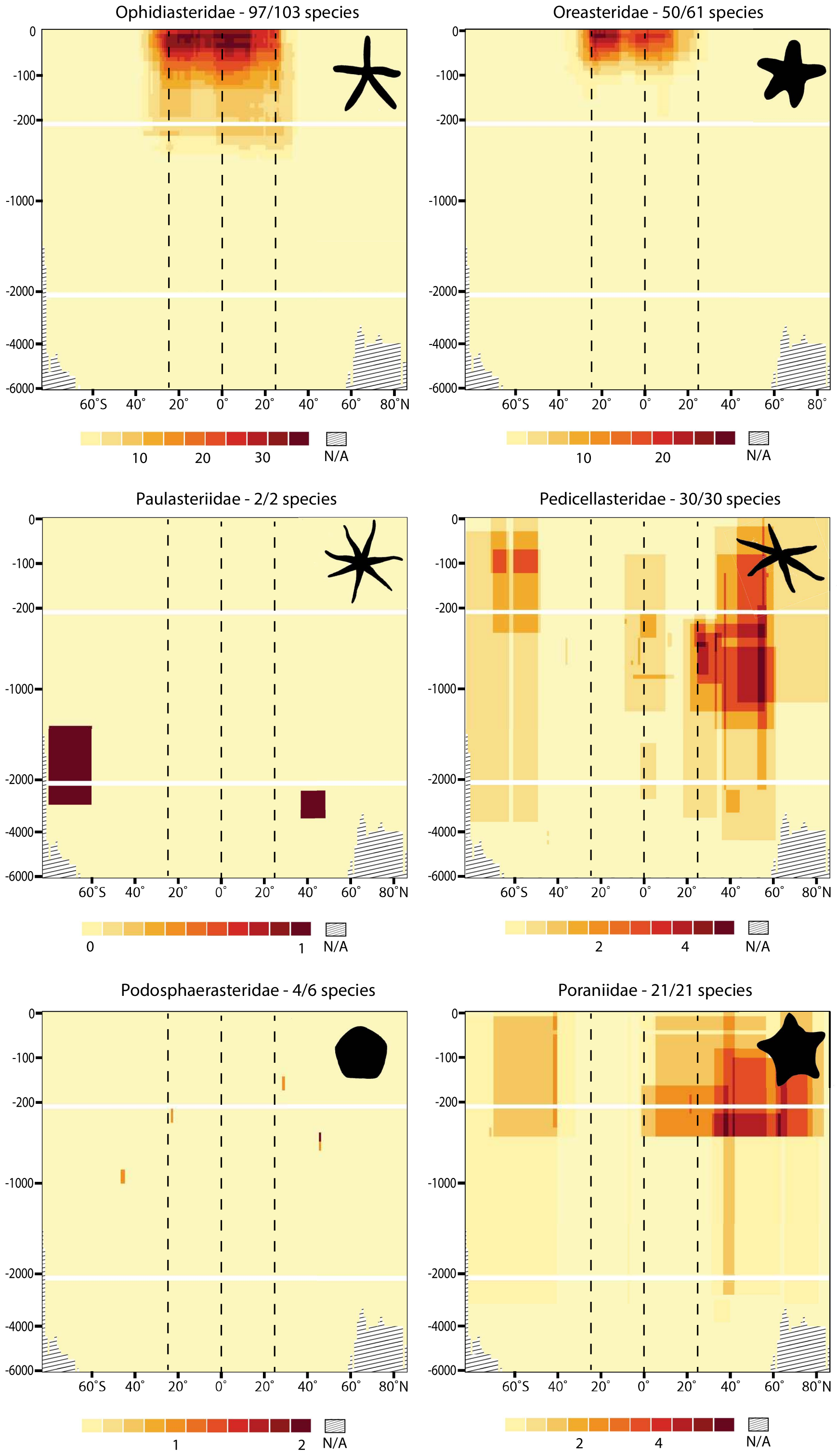
Ophidiasteridae - 97/103 species
Oreasteridae - 50/61 species
Paulasteriidae - 2/2 species
Pedicellasteridae - 30/30 species
Podosphaerasteridae - 4/6 species
Poraniidae - 21/21 species
0
-100
-200
-1000
-2000
-4000
-6000
60˚S
40˚
20˚
0˚
20˚
40˚
60˚
80˚N
10
20
30
N/A
0
1
2
4

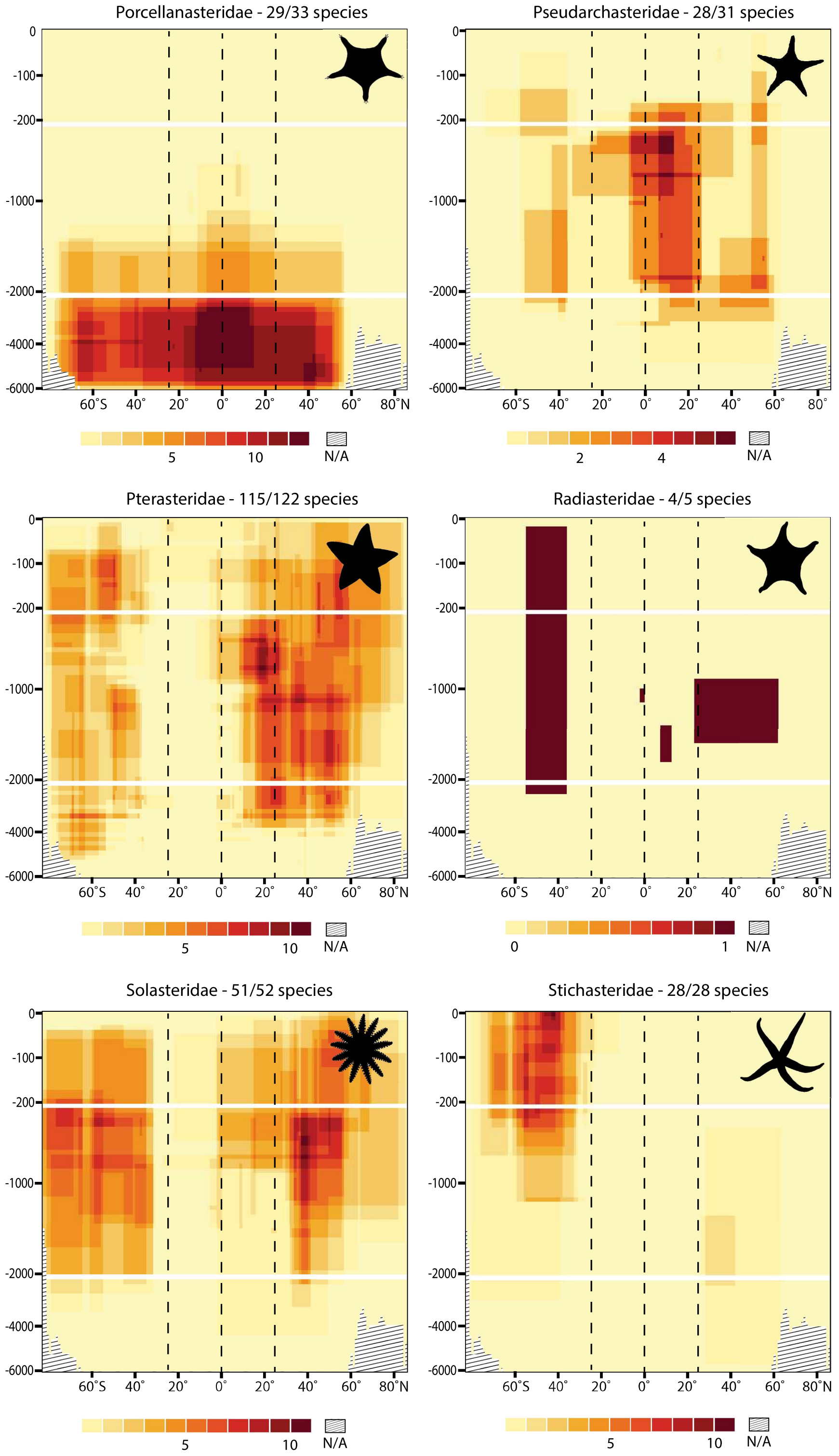

Porcellanasteridae - 29/33 species
Pseudarchasteridae - 28/31 species
Pterasteridae - 115/122 species
Radiasteridae - 4/5 species
Solasteridae - 51/52 species
Stichasteridae - 28/28 species
0
-100
-200
-1000
-2000
-4000
-6000
60°S
40°
20°
0°
20°
40°
60°
80°N
80°
5
10
2
4
0
1
N/A

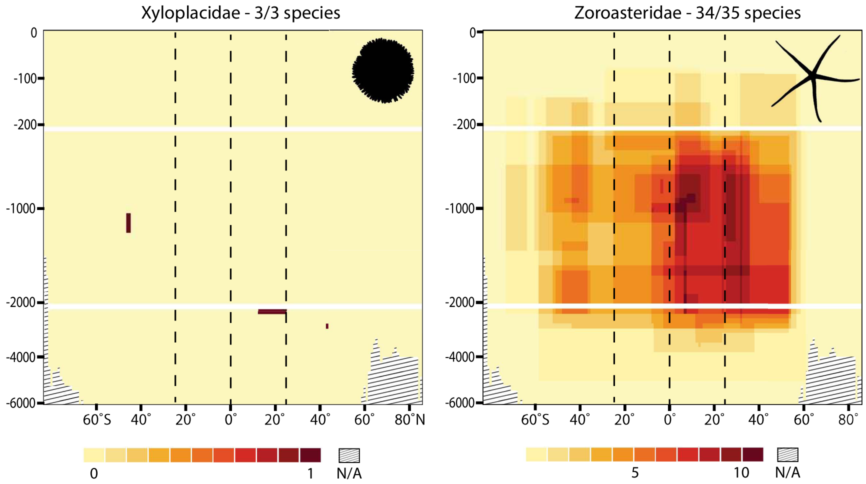


**Supplementary Figure 2:** Global bathy-diversity plots for all asteroid families. Most families (21/38) peak in diversity in the tropics with nine families (Acanthasteridae, Archasteridae, Asterinidae, Asterodiscidae, Asteropseidae, Luidiidae, Mithrodiidae, Ophidiasteridae and Oreasteridae) predominantly restricted to shallow tropical waters and a further two (Astropectinidae and Goniasteridae) extending into tropical upper bathyal waters. Eight families (Benthopectinidae, Brisingidae, Caymanostellidae, Freyellidae, Goniopectinidae, Porcellanasteridae, Pseudarchasteridae and Zoroasteridae) are most divers in bathyal or abyssal tropical waters with few, or no, shallow representatives. All are represented by at least some temperate taxa. Six families (Asteriidae, Echinasteridae, Pedicellasteridae, Poraniidae, Pterasteridae and Solasteridae) are most diverse anti-tropically in temperate waters. The Asteriidae, Echinasteridae and Poraniidae are most diverse at shallow temperate and polar latitudes while the Pedicellasteridae, Pterasteridae and Solasteridae are most diverse in deeper bathyal waters. All families have at least some representatives extending into tropical latitudes. A further four families (Ganeriidae, Heliasteridae, Odontasteridae and Stichasteridae) are most diverse in shallow and upper bathyal waters in the Southern Ocean, although all have representatives at lower latitudes. Graphical areas with no data because graph depth exceeds the deepest ocean depth at that latitude are indicated by grey and white dashed lines. Sampling effort for each family is indicated by two numbers: the number of species in our dataset and the total number of described species.

A

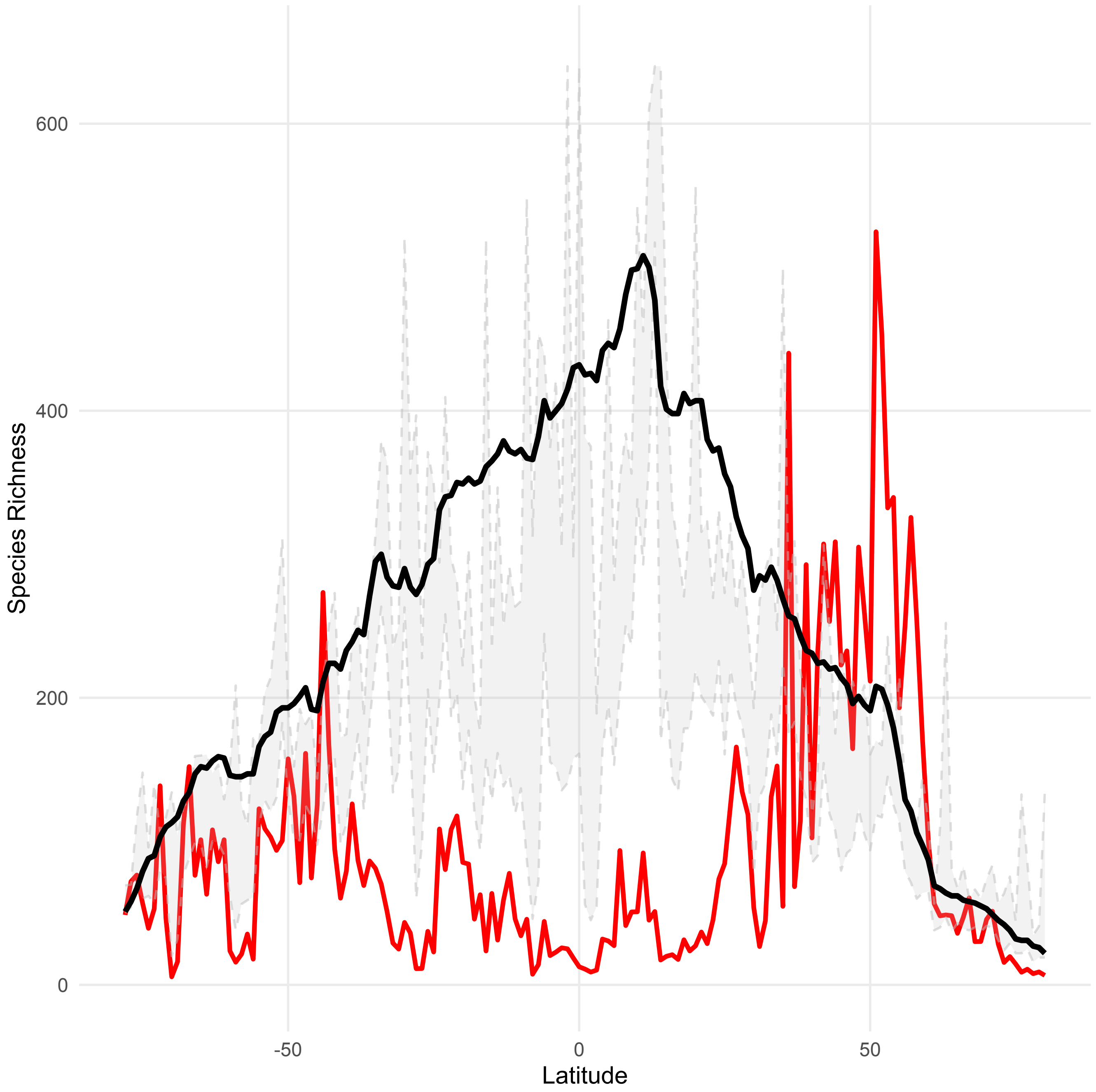

**B**

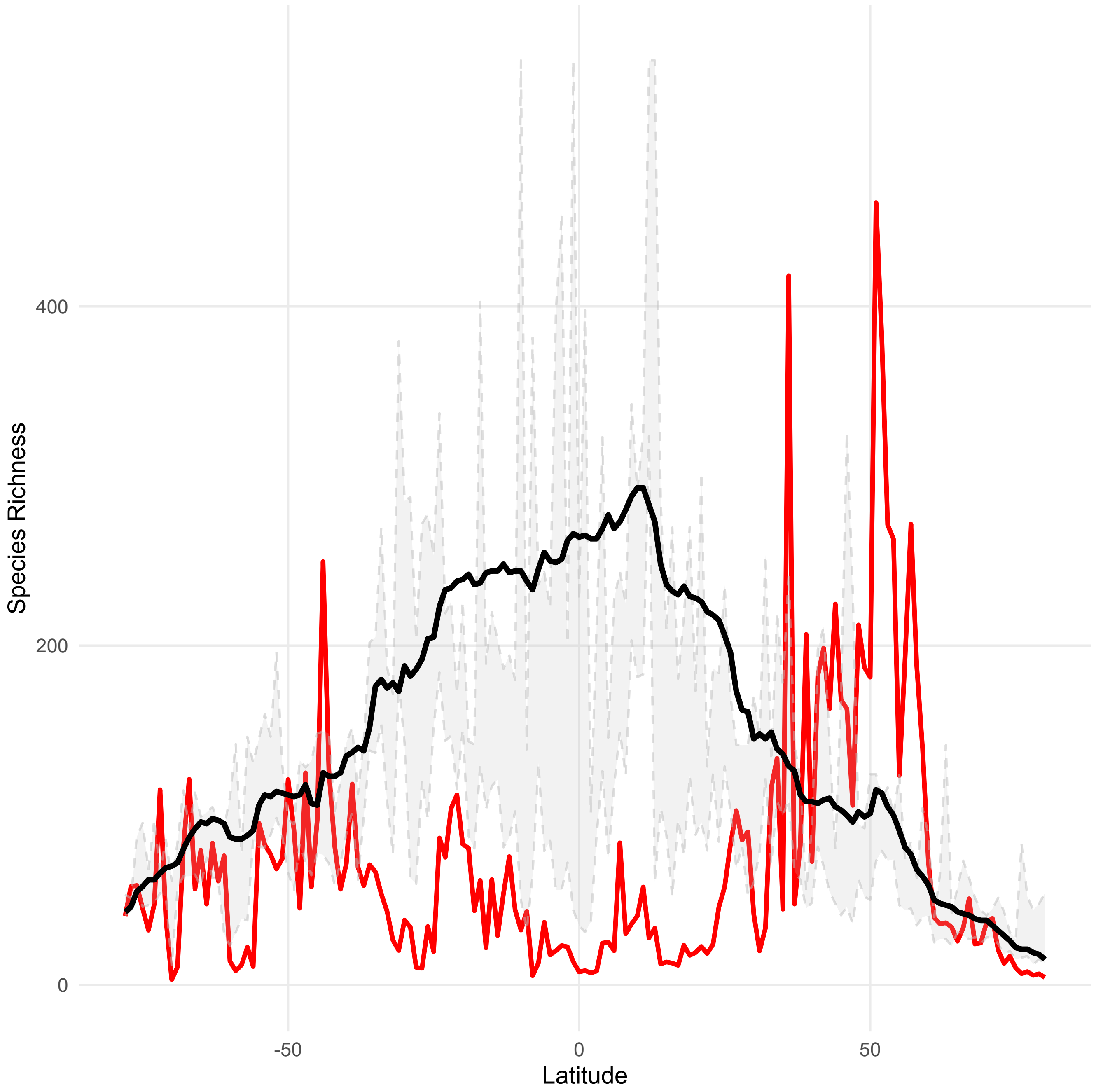

C

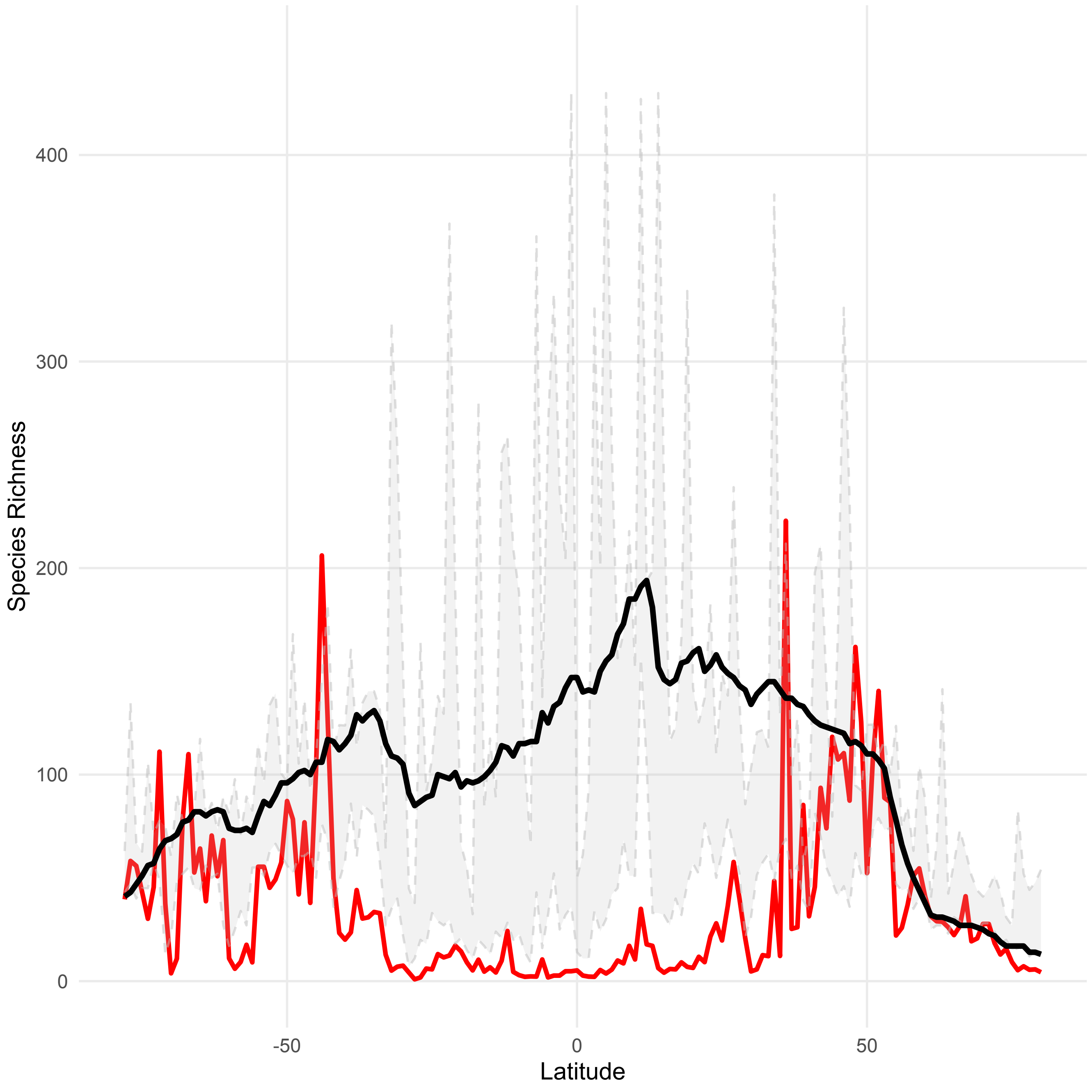

D

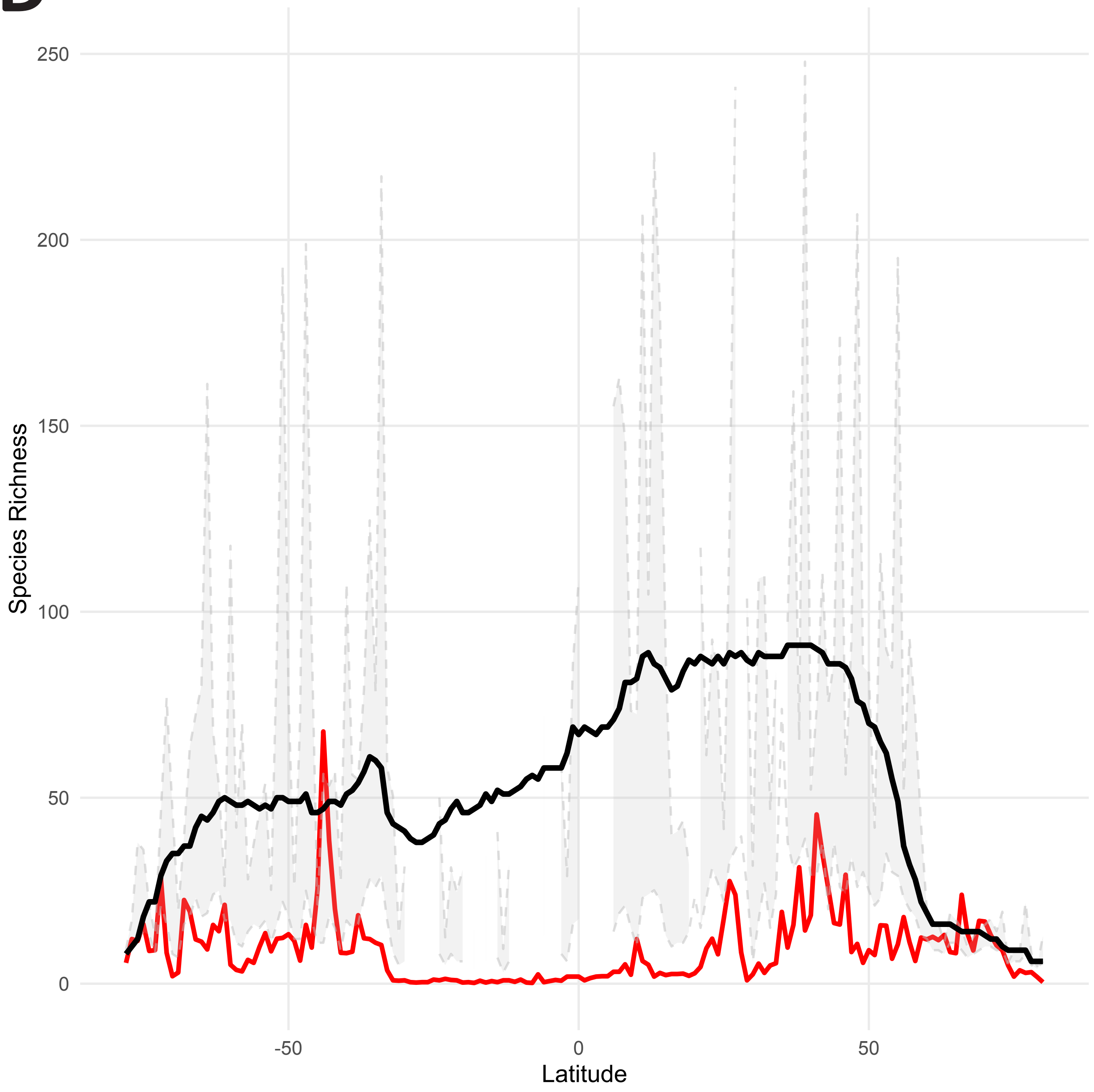


**Supplementary Figure 3:** Latitudinal Plots of Species Richness across three bathomes (Shallow: 0-200 m; Upper Bathyal: 200-2000 m; Lower Bathyal and Abyssal: 2000-6000 m), showing Informed Interpolated Richness (thick black line), Raw Richness (red line) and the upper and lower confidence interval for species richness at each degree of latitude calculated through use of Hill's Number calculations from raw presence/absence data (dashed interval). Analyese show that our interpolated values fall at the upper end, but generally within, these confidence intervals. **A:** Latitudinal richness plot across the full species occurrence dataset; **B:** Latitudinal richness plot across the shallow species occurrence dataset; **C:** Latitudinal richness plot across the upper bathyal species occurrence dataset; **D:** Latitudinal richness plot across the lower bathyal and abyssal species occurrence dataset.
